# Supplementary material for: Superspreading of SARS-CoV-2 in the USA
Source: PLoS One. 2021 Mar 25;16(3):e0248808. doi: 10.1371/journal.pone.0248808 (PMC7993775; doi:10.1371/journal.pone.0248808)
Supplement: S1 Appendix — (PDF) [file pone.0248808.s001.pdf]

Appendix S1: Simulations.

We employ Monte Carlo simulations to corroborate our theoretical calculations [Eqs (5) and (6)]. We start by adopting the conclusion of Ref. [A1] and defining $p(\beta)$ as a Gamma distribution with mean μ_β and standard deviation σ_β. We simulate an outbreak by first randomly generating a β from the distribution $p(\beta)$ and then drawing a random n from Pois$(n;\beta)$. This process is repeated until a non-zero n is generated, representing an outbreak starting in a given location with n individuals. Each of these n infected individuals is given a correspond infectiousness, β_i, which they keep for the remainder of the simulation. Each individual, i, then generates n_i new cases randomly drawn from Pois$(n_i;\beta_i)$, and each of these secondary infections is assigned its own randomly generated infectiousness as well. During every iteration of the simulation, representing a day, each infected individual infects others given by a new random Poisson variable with mean defined by their own infectiousness. After a set number of infections is reached, the simulation is stopped and the trajectory $I(t)$ is recorded. This process is repeated for 3,000 total trajectories, representing the ∼3,000 counties in the real USA data. This simulated data is then treated in the same manner as the real data, which is explained in "Data for COVID-19 in the USA" of the main text.

We also consider simulations with a recovery phase and a finite carrying capacity N. To implement recovery, we specify a given number of days, t_{rec}, over which an infected individual is infectious. Only those who contract the virus within this time period infect others. The effect of finite carrying capacity N is included by scaling the infectiousness β_i of individual i by the factor $S/N = 1-(I+R)/N$. For example, someone with infectiousness β_i generates a number of cases n_i drawn from the probability distribution Pois$(n;\beta_i(1-(I+R)/N))$ each day. This procedure is cut off once a certain fraction of N is reached, and then repeated 3,000 times. Although each trajectory follows the same $p(\beta)$, N can vary between different trajectories. To account for this variation in N, we normalize $\Delta I \to \sqrt{I}(\Delta I/I - \mu_\beta(1-(I+R)/N))$. We see that the simulated variance matches well with our theory (Fig 1).

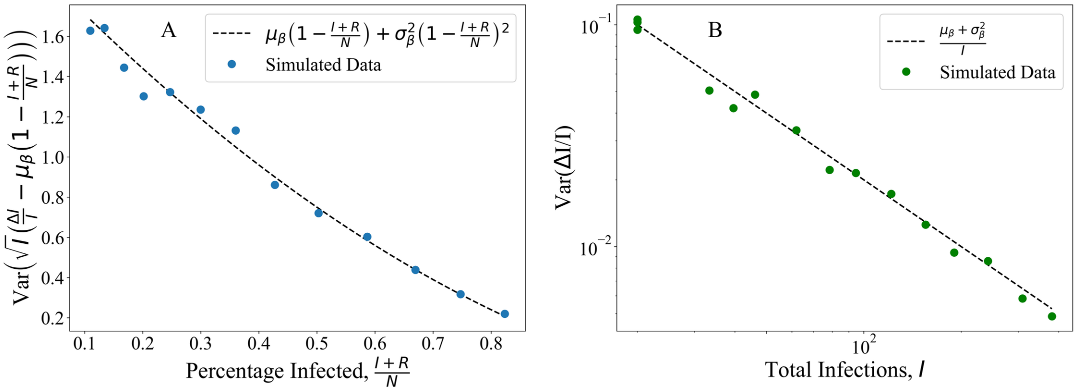

Fig 1. (a) Variance in the scaled growth rate for a simulated SIR model with various N values. The variance decreases as the susceptible population diminishes and infected individuals recover. (b) Simulation of an exponential model with no recovery, which is equivalent to early times in the pandemic. The variance in average infection rate starts at $\mu_\beta + \sigma_\beta^2$ at $I = 1$ and then decreases as $\sim 1/I$ as the number of infected individuals increases.

A References

[A1] Lloyd-Smith JO, Schreiber SJ, Kopp PE, Getz WM. Superspreading and the effect of individual variation on disease emergence. Nature. 2005;438:355–359.
