# Supplementary material for: Superspreading of SARS-CoV-2 in the USA
Source: PLoS One. 2021 Mar 25;16(3):e0248808. doi: 10.1371/journal.pone.0248808 (PMC7993775; doi:10.1371/journal.pone.0248808)
Supplement: S2 Appendix — (PDF) [file pone.0248808.s002.pdf]

Appendix S2: Variance in μ_β.

It is reasonable to question whether the calculated variance, σ_β^2, is a result of various geographic locations having differing average infectiousness, μ_β, due to varying population density, social norms, etc. One may instead consider that the mean infectiousness μ_β follows some distribution $q(\mu_\beta)$ among different counties. For a given μ_β, we have shown that the variance in $P(n;\mu_\beta,\sigma_\beta)$ averaged over I realizations is given by $\left(\mu_\beta^2+\sigma_\beta^2\right)/I$ in the exponential case. Including the effect of a distribution $q(\mu_\beta)$, we calculate the variance in $\Delta I/I$ to be:

$$\begin{aligned}
\mathrm{Var}\left(\frac{\Delta I}{I}\right) &= \sum_{n=0}^{\infty}(n-\bar{\mu}_\beta)^2\int_0^\infty d\mu_\beta\, q(\mu_\beta)P(n;\mu_\beta,\sigma_\beta)\\
&= \int_0^\infty d\mu_\beta\, q(\mu_\beta)\sum_{n=0}^{\infty}(n-\bar{\mu}_\beta)^2P(n;\mu_\beta,\sigma_\beta)\\
&= \int_0^\infty d\mu_\beta\, q(\mu_\beta)\left((\mu_\beta-\bar{\mu}_\beta)^2+\frac{\mu_\beta+\sigma_\beta^2}{I}\right)\\
&= \mathrm{Var}(q(\mu_\beta))+\frac{\bar{\mu}_\beta+\sigma_\beta^2}{I}
\end{aligned}$$

That is, when we account for the possibility that each region has a different μ_β, the value of μ_β is replaced by its mean $\bar{\mu}_\beta$ across counties, and a constant term is added for the variance in μ_β across counties. We can conclude that this variance cannot fully explain the data for two reasons. First, we observe a clear $\mathrm{Var}(\Delta I/I)\sim 1/I$ trend in the data (Fig 2), which can only be a result of the variance in $p(\beta)$ rather than $q(\mu_\beta)$. Additionally, we can directly measure the variance in μ_β across counties, which we find to be 0.007 (cases/day)^2. This number is too small to significantly affect the total variance in $\Delta I/I$, as seen in Fig 2. When the measured variance in $q(\mu_\beta)$ is taken into account in our fitting procedure, we find that $\bar{\mu}_\beta = 0.18$ cases/day, $\sigma_\beta \gtrsim 0.58$ cases^2/days^2, resulting in very slightly different value of $\sigma_\beta/\mu_\beta \gtrsim 3.1$.

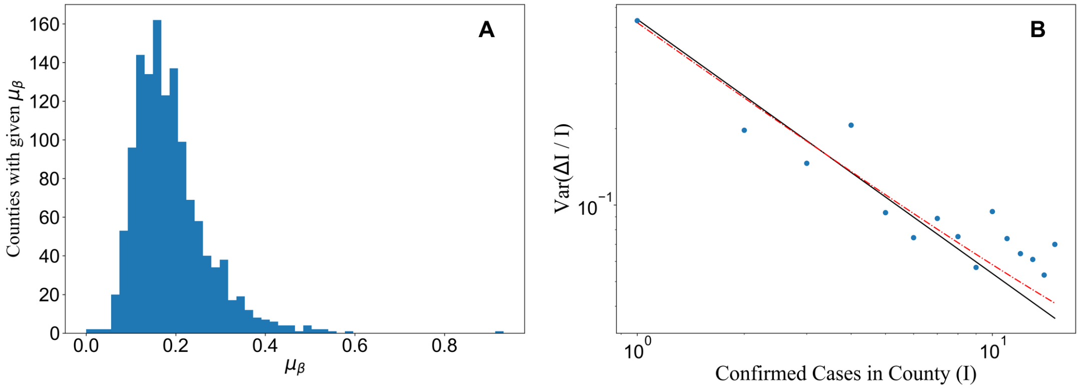

Fig 2. (a) The calculated value of the mean infectiousness, μ_β, for each individual county (with at least five cases). The variance in μ_β is relatively small: $\mathrm{Var}(\mu_\beta) = 0.0068$ (cases/day)$^2 \ll \sigma_\beta^2$. (b) When we account for this consideration (dashed red line), the fitted value of σ_β^2 decreases from 0.35 $\rightarrow$ 0.33 (cases/day)^2. This adjustment does not significantly affect our conclusions.
