# Supplementary material for: Superspreading of SARS-CoV-2 in the USA
Source: PLoS One. 2021 Mar 25;16(3):e0248808. doi: 10.1371/journal.pone.0248808 (PMC7993775; doi:10.1371/journal.pone.0248808)
Supplement: S3 Appendix — (PDF) [file pone.0248808.s003.pdf]

Appendix S3: Undetected cases.

Between asymptomatic cases and imperfect testing, there are a significant number of active cases, which can transmit the virus, that do not show up in the data set we use. One way to take this effect into account is by introducing an average probability that a case is detected, p_{det}. All variance calculations occur at fixed values of the number of detected cases, I_{det}. Given I_{det}, the probability that there are I total cases is given by a negative binomial distribution:

$$P(I; I_{\mathrm{det}}) = \binom{I-1}{I_{\mathrm{det}}-1} {p_{\mathrm{det}}}^{I_{\mathrm{det}}} (1-p_{\mathrm{det}})^{I-I_{\mathrm{det}}}. \tag{1}$$

If there are I active cases, then the probability that ΔI cases are generated is given by the sum of I random variables drawn from $P(n)$. Since $\mu_n = \mu_\beta$ and $\sigma_n^2 = \mu_\beta + \sigma_\beta^2$,

$$\sum_{\Delta I=0}^{\infty} P(\Delta I; I) \Delta I = \mu_\beta I \tag{2}$$

$$\sum_{\Delta I=0}^{\infty} P(\Delta I; I)(\Delta I - \mu_\beta I)^2 = (\mu_\beta + \sigma_\beta^2) I. \tag{3}$$

Once ΔI cases are generated on a given day, the probability that ΔI_{det} are detected is given by a binomial distribution:

$$P(\Delta I_{\mathrm{det}}; \Delta I) = \binom{\Delta I}{\Delta I_{\mathrm{det}}} {p_{\mathrm{det}}}^{\Delta I_{\mathrm{det}}} (1-p_{\mathrm{det}})^{\Delta I - \Delta I_{\mathrm{det}}}. \tag{4}$$

We combine these equations to derive the probability distribution for the number of new detected cases in a given day, ΔI_{det}, given that there are currently I_{det} active cases.

$$P(\Delta I_{\mathrm{det}}; I_{\mathrm{det}}) = \sum_{I=I_{\mathrm{det}}}^{\infty} P(I; I_{\mathrm{det}}) \sum_{\Delta I=\Delta I_{\mathrm{det}}}^{\infty} P(\Delta I; I) P(\Delta I_{\mathrm{det}}; \Delta I). \tag{5}$$

It follows that the mean and variance in $\Delta I_{\mathrm{det}}/I_{\mathrm{det}}$ are

$$\begin{aligned} \mathrm{Mean}\left(\frac{\Delta I_{\mathrm{det}}}{I_{\mathrm{det}}}\right) &= \frac{1}{I_{\mathrm{det}}} \sum_{\Delta I_{\mathrm{det}}=0}^{\infty} P(\Delta I_{\mathrm{det}}; I_{\mathrm{det}}) \Delta I_{\mathrm{det}} \\ &= \mu_\beta \end{aligned} \tag{6}$$

$$\begin{aligned} \mathrm{Var}\left(\frac{\Delta I_{\mathrm{det}}}{I_{\mathrm{det}}}\right) &= \frac{1}{I_{\mathrm{det}}^2} \sum_{\Delta I_{\mathrm{det}}=0}^{\infty} P(\Delta I_{\mathrm{det}}; I_{\mathrm{det}}) (\Delta I_{\mathrm{det}} - I_{\mathrm{det}}\mu_\beta)^2 \\ &= \frac{\mu_\beta + \mu_\beta^2(1-p_{\mathrm{det}}) + p_{\mathrm{det}}\sigma_\beta^2}{I_{\mathrm{det}}}. \end{aligned} \tag{7}$$

That is, when under-detection is accounted for, an extra term $\mu_\beta^2(1-p_{\mathrm{det}})/I_{\mathrm{det}}$ is added to the variance due to the variance in the underlying total number of cases, I. The term $\sigma_\beta^2/I_{\mathrm{det}}$ is also scaled down by a factor p_{det}, since there are on average a larger number $I \sim I_{\mathrm{det}}/p_{\mathrm{det}}$ of total cases. Thus, since $\mu_\beta^2(1-p_{\mathrm{det}}) \ll \mu_\beta$ and σ_β^2 is suppressed by a factor of p_{det}, the calculated value of σ_β^2 is a lower bound.

Although there are estimates of the fraction of detected COVID-19 cases in literature (e.g., Refs. [C1–C3]), in order to be conservative we do not directly use these estimates for the parameter p_{det} in Eq (7), due to the possibility that undetected cases have a different (likely lower) infectiousness.

C References

[C1] Pedersen M, Meneghini M. Quantifying undetected COVID-19 cases and effects of containment measures in Italy: Predicting phase 2 dynamics. 2020;doi:10.13140/RG.2.2.11753.85600.

[C2] Li R, Pei S, Chen B, Song Y, Zhang T, Yang W, et al. Substantial undocumented infection facilitates the rapid dissemination of novel coronavirus (SARS-CoV-2). Science. 2020;368(6490):489–493. doi:10.1126/science.abb3221.

[C3] Lu FS, Nguyen AT, Link NB, Lipsitch M, Santillana M. Estimating the Early Outbreak Cumulative Incidence of COVID-19 in the United States: Three Complementary Approaches. medRxiv. 2020; p. 2020.04.18.20070821. doi:10.1101/2020.04.18.20070821.
