# Supplementary material for: Superspreading of SARS-CoV-2 in the USA
Source: PLoS One. 2021 Mar 25;16(3):e0248808. doi: 10.1371/journal.pone.0248808 (PMC7993775; doi:10.1371/journal.pone.0248808)
Supplement: S4 Appendix — (PDF) [file pone.0248808.s004.pdf]

Appendix S4: Variance in testing.

We have shown that a fixed detection rate, p_{det}, across counties cannot account for the variance observed within the US population. However, one can also check to ensure that variation in p_{det} between counties, described by a probability distribution $q(p_{\mathrm{det}})$, does not explain the data either. To account for differing values of p_{det} we weight Eq (4) by $q(p_{\mathrm{det}})$ so that $P(\Delta I_{\mathrm{det}}; \Delta I) \rightarrow \int_0^1 dp_{\mathrm{det}} q(p_{\mathrm{det}}) P(\Delta I_{\mathrm{det}}; \Delta I)$. Plugging into Eg. (7) we see

$$\mathrm{Var}\left(\frac{\Delta I_{\mathrm{det}}}{I_{\mathrm{det}}}\right) = \frac{\mu_\beta + \mu_\beta^2(1 - \overline{p_{\mathrm{det}}}) + \overline{p_{\mathrm{det}}}\sigma_\beta^2}{I_{\mathrm{det}}} \tag{8}$$

where $\overline{p_{\mathrm{det}}}$ is the mean detection rate across all counties when they have I_{det} cases. This expression shows that the variance in the exponential growth rate $(\Delta I_{\mathrm{det}})/I_{\mathrm{det}}$ only depends on the mean detection rate at a given I_{det} rather than its variance. Furthermore, averaging p_{det} across counties at various times (but the same I_{det}) will average out any effects from cyclical weekly reporting patterns. To observe how this impacts our calculation for σ_β^2, we rearrange Eq. (8) to obtain:

$$\sigma_\beta^2 = \frac{\mathrm{Var}\left(\frac{\Delta I_{\mathrm{det}}}{I_{\mathrm{det}}}\right) I_{\mathrm{det}} - \mu_\beta - \mu_\beta^2(1 - \overline{p_{\mathrm{det}}})}{\overline{p_{\mathrm{det}}}} = \frac{\sigma^2_{\beta, p_{\mathrm{det}}=1} - \mu_\beta^2(1 - \overline{p_{\mathrm{det}}})}{\overline{p_{\mathrm{det}}}} \tag{9}$$

where $\sigma^2_{\beta, p_{\mathrm{det}}=1}$ is the variance we calculate in the main text assuming $\overline{p_{\mathrm{det}}} = 1$. Since $\mu_\beta^2(1 - \overline{p_{\mathrm{det}}}) \ll \sigma^2_{\beta, p_{\mathrm{det}}=1}$, it is clear that accounting for a imperfect detection rate can only increase the variance in infectiousness. Therefore, if $\overline{p_{\mathrm{det}}} < 1$ this makes our calculation a lower bound on σ_β^2. Further, if we use the percentage of asymptomatic cases, 40% [D1], as a rough estimate for the mean percentage of undetected cases, then μ_β remains unchanged while σ_β increases from 0.59 cases/day to 0.75 cases/day. This change in the variance corresponds to a significant increase in superspreading as the percentage of new infections cause by the top 5% of infectious cases rises from 61.7% to 74.0%. While this exercise provides some insight into how large σ_β could be, it is not a rigorous upper bound. Firstly, there remains significant uncertainty in the percentage of asymptomatic cases as estimates range from 8.2% to 75% [D2]. Additionally, there remain other complications, such as incubation period variation and cross-county interactions, which would increase the variance further.

D References

[D1] COVID-19 Pandemic Planning Scenarios. CDC. 2020;https://www.cdc.gov/coronavirus/2019-ncov/hcp/planning-scenarios-h.pdf.

[D2] Yanes-Lane M, Winters N, Fregonese F, Bastos M, Perlman-Arrow S, Campbell JR, et al. Proportion of asymptomatic infection among COVID-19 positive persons and their transmission potential: A systematic review and meta-analysis. PLOS ONE. 2020;15(11):1–21. doi:10.1371/journal.pone.0241536.
