# Supplementary material for: Superspreading of SARS-CoV-2 in the USA
Source: PLoS One. 2021 Mar 25;16(3):e0248808. doi: 10.1371/journal.pone.0248808 (PMC7993775; doi:10.1371/journal.pone.0248808)
Supplement: S5 Appendix — (PDF) [file pone.0248808.s005.pdf]

Appendix S5: Cross-county interactions.

Our analysis in the main text operates under the assumption that each county in the USA is an independent population in which the virus can spread. However, it is clear that there is some portion of infections that cross county lines. To understand how this interaction can affect the variance in observed growth rate of cases, we explore what the variance looks like if we have perfect mixing between M counties, each with I_1 active cases. In this formulation, the variance we calculate is $\mathrm{Var}(\Delta I_1/I_1)$ at a given I_1. Focusing on a single county with I_1 active cases, the probability that there are I total cases across the M counties is given by a negative binomial distribution:

$$P(I; I_1) = \binom{I-1}{I_1-1} \left(\frac{1}{M}\right)^{I_1} \left(1 - \frac{1}{M}\right)^{I-I_1}. \tag{10}$$

We have shown that if there are I total cases, then the mean and variance in the number of new cases, ΔI, is $\mu_\beta I$ and $(\mu_\beta + \sigma_\beta^2)I$, respectively. Once ΔI cases are generated, they are randomly sorted into the M counties. Therefore,

$$P(\Delta I_1; \Delta I) = \binom{\Delta I}{\Delta I_1} \left(\frac{1}{M}\right)^{\Delta I_1} \left(1 - \frac{1}{M}\right)^{\Delta I - \Delta I_1}. \tag{11}$$

Combining these distributions, the probability that ΔI_1 cases occur in a given county that has I_1 active cases is:

$$P(\Delta I_1; I_1) = \sum_{I=I_1}^{\infty} P(I; I_1) \sum_{\Delta I = \Delta I_1}^{\infty} P(\Delta I; I) P(\Delta I_1; \Delta I). \tag{12}$$

From $P(\Delta I_1; I_1)$ we calculate the mean and variance in $\Delta I_1/I_1$ to find:

$$\begin{aligned} \mathrm{Mean}\left(\frac{\Delta I_1}{I_1}\right) &= \frac{1}{I_1} \sum_{\Delta I_1 = 0}^{\infty} P(\Delta I_1; I_1) \Delta I_1 \\ &= \mu_\beta \end{aligned} \tag{13}$$

$$\begin{aligned} \mathrm{Var}\left(\frac{\Delta I_1}{I_1}\right) &= \frac{1}{I_1^2} \sum_{\Delta I_1 = 0}^{\infty} P(\Delta I_1; I_1) (\Delta I_1 - I_1 \mu_\beta)^2 \\ &= \frac{\mu_\beta + \mu_\beta^2 \left(1 - \frac{1}{M}\right) + \frac{\sigma_\beta^2}{M}}{I_1}. \end{aligned} \tag{14}$$

Since $\mu_\beta = 0.18$ cases/day, the term $\mu_\beta + \mu_\beta^2(1 - 1/M)$ cannot account for the variation present in the US. Therefore, if there is maximal interactions between counties, then the calculation of σ_β^2 remains a lower bound estimate. Intuitively, one can say that when different counties interact strongly with each other, there is a larger underlying number of active cases from which new cases can be drawn for a given county, and this larger number reduces the statistical variance.

The previous consideration assumes that all counties interact evenly with each other. It is possible that some counties might gain a large number of cases entering from a neighboring county, while others have more exiting than entering. To account for this potential source of variance we consider a single county. Assume there is a fixed portion, p_{exit}, of new infections from cases within a county that are spread to an outside county, as well as a portion, p_{enter}, of new cases from other counties. Both of these quantities are defined in terms of the number of cases in the current county, $I(t)$. Consequently, the effective number of cases leading to new infections in the current county is $(1 - p_{\mathrm{exit}} + p_{\mathrm{enter}})I(t)$. Since all counties are assumed to follow the same underlying distribution $p(\beta)$, we expect that the mean of $\Delta I/I$ will be $(1 - p_{\mathrm{exit}} + p_{\mathrm{enter}})\mu_\beta$. With this understanding, there are two possibilities: either all counties have equal flows in and out so that $p_{\mathrm{exit}} \sim p_{\mathrm{enter}}$, or there is a balance between counties with $p_{\mathrm{exit}} > p_{\mathrm{enter}}$ and vice versa. In the first case, we see that all

counties share approximately the same measured μ_β while in the later, there is a wide spread in μ_β from this cross county interaction. Since we show in Appendix S2 that the there is little variance in μ_β across counties, this suggests that $p_{\text{exit}} \sim p_{\text{enter}}$ within each county. Consequently, the effective number of cases that can lead to a new case within a given county, $(1 - p_{\text{exit}} + p_{\text{enter}})I(t)$, varies from the true number, $I(t)$, only on the order of $\sigma_{\mu_\beta} \ll \sigma_\beta$. Therefore, we conclude that this effect cannot explain the large variance we observe.
