# Supplementary material for: Superspreading of SARS-CoV-2 in the USA
Source: PLoS One. 2021 Mar 25;16(3):e0248808. doi: 10.1371/journal.pone.0248808 (PMC7993775; doi:10.1371/journal.pone.0248808)
Supplement: S6 Appendix — (PDF) [file pone.0248808.s006.pdf]

Appendix S6: Variance in incubation period.

Our analysis ignores the effect of latency between the moment of infection and the appearance of symptoms that can lead to detection, often called the incubation period. This treatment is equivalent to assuming that there is a fixed incubation period for all infections. In this situation, if the true number of cases at a given time (counting cases as those infected, and not necessarily already detected) is $I(t)$, then the number of cases observed is simply $I_{\text{observed}}(t) = I(t-L)$, where L is the amount of time for an individual to start showing symptoms and be detected. However, it is reasonable to suspect that some individuals could have shorter or longer incubation periods, and this variance could impact the overall variance that we calculate. To address this, we assume that the duration of incubation time, L, follows some distribution $q(L)$ with mean μ_L. At a given time t, the infections being observed are originating from times in the vicinity of $t - \mu_L$. Therefore, the effective number of infected individuals leading to new infections at time t is $\sum_{L=\mu_L-\Delta L}^{\mu_L+\Delta L} q(L)I(t-L)$, where $2\Delta L$ represents the range of possible incubation periods. Since $I(t)$ grows exponentially, the terms from the most recent time with smaller incubation period, $L = \mu_L - \Delta L$, will dominate the sum. Consequently, the effective infected population is always greater than or equal to $I(t - \mu_L)$, and this effect tends to decreases the overall variance in $\Delta I/I$. This diminishing of the variance with increasing ΔL is corroborated by simulations (Fig 3).

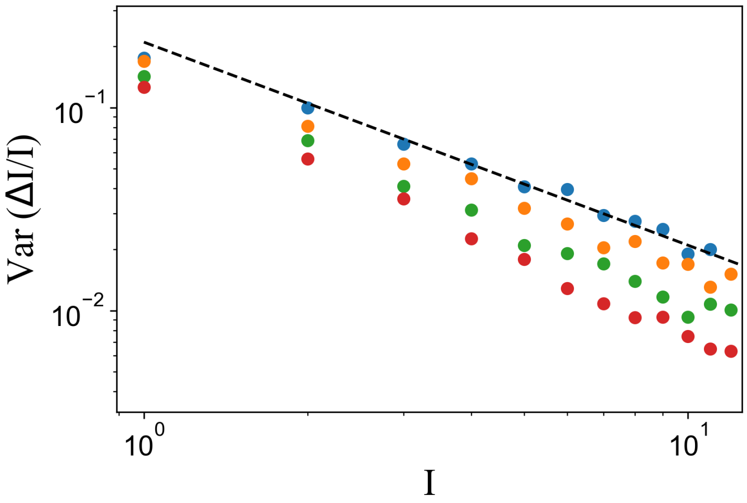

Fig 3. We simulate the impact of variance in incubation time compared to a fixed incubation period (blue). Here, we use $p(L) \sim e^{-\lambda L}$ with $\lambda = 1, 2, 3$ (orange, green, red). Variance in incubation time only decreases the observed variance and consequently cannot explain the large σ_β we calculate.
