# Supplementary material for: Superspreading of SARS-CoV-2 in the USA
Source: PLoS One. 2021 Mar 25;16(3):e0248808. doi: 10.1371/journal.pone.0248808 (PMC7993775; doi:10.1371/journal.pone.0248808)
Supplement: S7 Appendix — (PDF) [file pone.0248808.s007.pdf]

Appendix S7: Dispersion parameter comparison.

The dispersion parameter is typically defined as $\mu_{R_0}^2/\sigma_{R_0}^2$, where μ_{R_0} and $\sigma_{R_0}^2$ are the mean and variance of the number of new infections caused by an individual over their entire infectious period. Since we work with the daily infection rate, β, and calculate $\mu_\beta^2/\sigma_\beta^2$ it is important to draw a direct comparison between these two dispersion parameters.

We can understand the distinction between $\mu_\beta^2/\sigma_\beta^2$ and $\mu_{R_0}^2/\sigma_{R_0}^2$ using the SIR-based framework that we establish in the main text. This approach involves calculating the mean (μ_{R_0}) and variance ($\sigma_{R_0}^2$) of R_0, the number of infections caused by an infected individual over the course of their infection.

First, if we assume a fixed infection period of D days, then we see that the total number of infections from an individual with infectiousness β is the sum of D draws form a Poisson distribution with mean β. Therefore,

$$p(R_0;\beta) = \frac{e^{-D\beta}(D\beta)^{R_0}}{R_0!}.$$

Thus across the entire population, with infectiousness described by an arbitrary distribution $p(\beta)$, the distribution of R_0 is simply $p(R_0) = \int_0^\infty d\beta p(\beta)p(R_0;\beta)$. From this distribution we can calculate the mean and variance of R_0:

$$\begin{aligned}
\mu_{R_0} &= \sum_{R_0=0}^{\infty} p(R_0)R_0 \\
&= \int_0^\infty d\beta p(\beta)e^{-D\beta}\sum_{R_0=0}^{\infty}\frac{(D\beta)^{R_0}}{R_0!}R_0 \\
&= \int_0^\infty d\beta p(\beta)D\beta \\
\mu_{R_0} &= D\mu_\beta
\end{aligned}$$

$$\begin{aligned}
\sigma_{R_0}^2 &= \sum_{R_0=0}^{\infty} p(R_0)(R_0-\mu_{R_0})^2 \\
&= \int_0^\infty d\beta p(\beta)e^{-D\beta}\sum_{R_0=0}^{\infty}\frac{(D\beta)^{R_0}}{R_0!}(R_0-D\mu_\beta)^2 \\
&= \int_0^\infty d\beta p(\beta)\left[D^2(\beta-\mu_\beta)^2+D\beta\right] \\
\sigma_{R_0}^2 &= D^2\sigma_\beta^2 + D\mu_\beta
\end{aligned}$$

Combining these results we see that the usual dispersion parameter is given by

$$\frac{\mu_{R_0}^2}{\sigma_{R_0}^2} = \frac{\mu_\beta^2}{\sigma_\beta^2+\frac{\mu_\beta}{D}}.$$

From this expression, it becomes apparent that while $\mu_{R_0}^2/\sigma_{R_0}^2$ and $\mu_\beta^2/\sigma_\beta^2$ are not exactly equivalent, in our case they are very nearly equal since $\sigma_\beta^2 \gg \mu_\beta/D$. If we let $D = 14$ days and calculate the duration of infection dispersion parameter we obtain $\mu_{R_0}^2/\sigma_{R_0}^2 = 0.093$, as compared to our value of 0.096.

One can be more careful and let the infectious period be described by an arbitrary distribution $p(D)$ with mean μ_D and variance σ_D^2. With this setup, the duration of infection dispersion gains an extra term in the denominator:

$$\frac{\mu_{R_0}^2}{\sigma_{R_0}^2} = \frac{\mu_\beta^2}{\sigma_\beta^2 + \left(\frac{\sigma_D}{\mu_D}\right)^2 (\mu_\beta^2 + \sigma_\beta^2) + \frac{\mu_\beta}{\mu_D}}.$$

If we use $\mu_D = 13.4$ days [G1] and conservatively estimate $\sigma_D \sim 5$ days, then the dispersion parameters remain almost unchanged.

It is worth emphasizing that the primary result of our analysis, the Lorenz curve relationship (Eq. (9) and Fig. 3 of the main text), does not depend on the difference between these two definitions of the dispersion parameter. The Lorenz curve relation is based on the ratio $\mu_\beta^2/\sigma_\beta^2$, and allows one to obtain results for the percentage of cases caused by (say) the 20% of most infectious cases. Our results for the Lorenz curve are consistent with other studies, while providing more quantitative detail.

G References

[G1] Byrne AW, McEvoy D, Collins AB, Hunt K, Casey M, Barber A, et al. Inferred duration of infectious period of SARS-CoV-2: rapid scoping review and analysis of available evidence for asymptomatic and symptomatic COVID-19 cases. BMJ Open. 2020;10(8). doi:10.1136/bmjopen-2020-039856.
