# Supplementary material for: Superspreading of SARS-CoV-2 in the USA
Source: PLoS One. 2021 Mar 25;16(3):e0248808. doi: 10.1371/journal.pone.0248808 (PMC7993775; doi:10.1371/journal.pone.0248808)
Supplement: Attachment — Submitted filename: Response to Reviewers.pdf [file pone.0248808.s008.pdf]

Dear Editors,

We thank you for your continued consideration of our manuscript, "Superspreading of SARS-CoV-2 in the USA". We also thank the reviewers for their detailed comments, which have led us to make significant improvements in the clarity and thoroughness of our original manuscript. In the previous round of review, Reviewer 1 expressed concern about the impact of the variance in incubation periods on our result, and also suggested applying our method to previous, well understood epidemics. Reviewer 2 outlined several considerations, including the distinction between the dispersion parameter based on infection rate and the dispersion parameter based on duration of infection, the ability to obtain a range or upper bound for our analysis, as well as the impacts of weekly reporting patterns and variance in change of detection rate on our result. The reviewer also provided more minor suggestions. Below we list the full content of the reports from both reviewers, and we respond to all of their comments systematically. With these revisions we consider our manuscript ready for publication in PLoS ONE.

Reviewer 2 Comment:

[This comment has been moved to the top to avoid confusion on the switch in notation.] The choice of k to denote the transmission rate seem to be a likely source of confusion as k is frequently used as the dispersion parameter in literature on superspreading (e.g., see refs 10, 16, 18 from the manuscript).

Response:

We have switched our notation for the infectiousness from k to β. As reviewer 2 aptly notes, this switch will limit confusion with the canonical use of k for the dispersion parameter. Additionally, the change is consistent with the notation typically used for the infectiousness in SIR models.

Reviewer 1 Comment:

In this study, Calvin and colleagues introduced a novel method to determine the variation of infectiousness among populations based on SIR model. And then authors used this method to illustrate there was significant variations of infectiousness among populations in USA known as superspreading events. The study is interesting and the result (superspreading events in USA) will also contribute to tailor the prevention and control policies for COVID-19 epidemic in USA. However, I have several concerns about this study.

Response:

Reviewer 1 provides a summary on our original manuscript outlining the main result of our paper. We thank them for their time and comprehensive comments.

Reviewer 1 Comment:

The COVID-19 has highly varied incubation period (with mean of 5.2 days, and the distribution of incubation period was estimated as 12.5 days). The highly variable incubation time would result in surge for the number of confirmed cases in some specific times. As the method only used the number of confirmed case each day, it is important to know how the highly variable incubation time affect the result?

Response:

Reviewer 1 raises an important point regarding the impact of incubation period on the results of our study. This concern, which we refer to as latency, is addressed in Appendix S6 of the original submission. In order to avoid any confusion with terminology in the literature, we have edited our manuscript and appendix to refer to this consideration as incubation period. In Appendix S6, we argue that variance in infectious period would only result in a larger σ_β^2. We make a heuristic argument based on the increased effective population given the current number of confirmed cases, and we also provide numeric simulations with increasing variation in incubation period. We see that increasing the variance in the incubation period only leads to a decrease in the variance of $\Delta I/I$. Consequently, since we know that there is considerable variance in the incubation period, the variance we observe is a lower bound.

Reviewer 1 Comment:

As a novel method, it should be first tested in previous data (such as SARS-CoV, Ebola, ZIKA etc) to illustrate the accuracy of the method. Then the result for COVID-19 would be more convincing.

Response:

The suggestion by Reviewer 1 to test our method on previous epidemic data with well established dispersion parameters is a reasonable recommendation. The difficulty with this suggestion is that the publicly available data for previous epidemics generally does not have sufficient depth and level of resolution to perform the analysis we suggest and arrive at a confident conclusion. Nonetheless, we have attempted to apply our method using data from the SARS-CoV-1 epidemic in 2003 (available here). The results presented below highlight the difficulties.

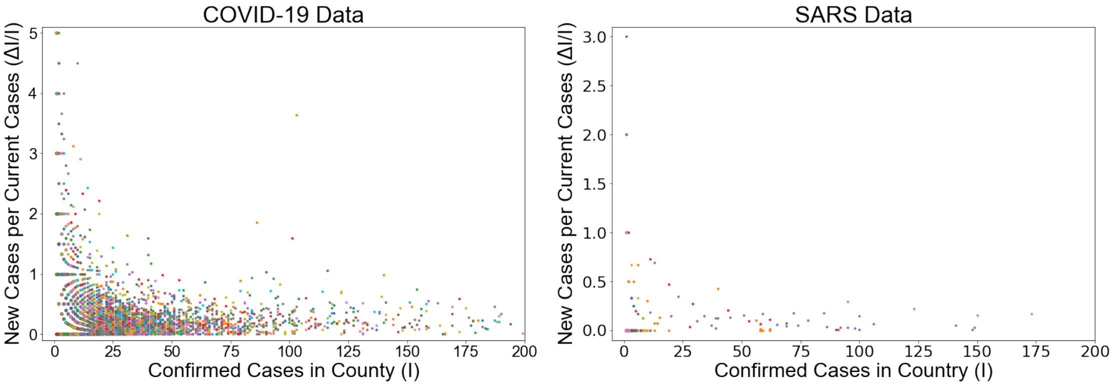

Figure 1: Comparison of US COVID-19 data to SARS data across several countries. In both cases, we observe a similar shrinking envelope as the number of confirmed cases increases. However, it is apparent that COVID-19 data is far more robust due to a larger number of regional subdivisions with substantial numbers of cases.

We begin by taking the early time confirmed cases trajectories for the smallest division available in the data, which is at the country level. Since our analysis assumes that each region has the same $p(\beta)$, using a data set this coarse grained is rather tenuous, however it was the most detailed, publicly available data we could find. When we attempt our analysis with this data, we see that the variance in new cases per current cases decreases as the number of cases increases, but the data is much scarcer than COVID-19 data, as shown in Fig. 1 below.

Continuing with this data, we can find the variance in $\Delta I/I$ at various I values with sufficient number of occurrences. With the COVID-19 data, we included only values of I with at least 250 occurrences. For

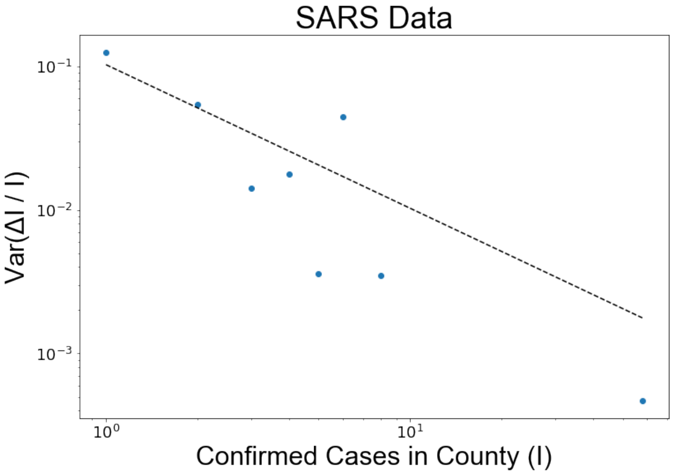

Figure 2: Decrease in variance of new cases per confirmed cases in SARS data. As the number of cases increases, the variance decreases by the central limit theorem.

the SARS data we need to decreases the cut-off to 5 occurrences to get even 8 values. These data points are shown, with their fit, in Fig. 2.

Through this analysis, we obtain a mean infectiousness of $\mu_\beta = 0.065$ infections per day and standard deviation of $\sigma_\beta = 0.19$ infections per day. When we convert this to a duration of infection dispersion parameter we get 0.102 which is qualitatively consistent with literature values of 0.16 (90% CI: 0.11-0.64) in Singapore and 0.17 (90% CI: 0.10-0.64) in Beijing (https://www.nature.com/articles/438293a).

While this result is consistent with more in-depth studies, it is important to note that our analysis should not be considered robust on a data set with such limited subdivision over vastly different geographic regions. In order to safely perform our analysis, we would need the data in a given region to be separated into hundreds of sub-regions. While the reviewer's suggestion remains a good one and would be very instructive if possible, the data is unfortunately not available to soundly carry it out.

Reviewer 2 Comment:

In this article, the authors derive a relationship between the variance in epidemic growth rates and current size of the epidemic, formalizing the intuition that super spreading has the greatest impact on epidemic dynamics when the number of infected individuals is small. They then apply this relationship to COVID-19 case data from the U.S. to estimate superspreading of SARS-CoV-2.

Response:

Reviewer 2 provides a summary on our original manuscript outlining the main result of our paper. We thank them for their time and extensive comments.

Reviewer 2 Comment:

The authors compare their estimate of μ_k^2/σ_k^2 base on mean and standard deviation of daily transmission rate to the dispersion parameters presented in the literature which are instead based on duration of infection transmission rate. These numerical estimates therefore seem incomparable without further transformation. For example, if the daily transmission rate was Gamma distributed with mean μ_k and variance σ_k^2 and each individual's daily transmission rate was a independent draw from this distribution, I believe their duration

of infection transmission rate (based on the manuscript's assumption of a 14 day duration of infection) would have a distribution with the ratio of mean^2/variance being $14\mu_k^2/\sigma_k^2$.

Response:

Reviewer 2 brings up an important distinction between the dispersion parameter for the daily transmission rate and the duration of infection transmission rate. Indeed, as the reviewer notes, the dispersion parameter reported in literature is typically based on the total number of infections R_0 rather than on the daily infection rate β, and it is important for us to explicitly draw a relation between these two quantities in order to make a fair comparison. Fortunately, it is possible to explicitly derive a relation between these two quantities, $\mu_\beta^2/\sigma_\beta^2$ and $\mu_{R_0}^2/\sigma_{R_0}^2$. The relation shows that these two metrics are largely equivalent, especially in an overdispersed case. In order to clarify this important distinction, we have added a note explaining this point, which has been added as reference 34 in the manuscript. In this letter we provide some more detailed commentary.

We can understand the distinction between $\mu_\beta^2/\sigma_\beta^2$ and $\mu_{R_0}^2/\sigma_{R_0}^2$, using the SIR-based framework that we establish in the paper. This involves calculating the mean (μ_{R_0}) and variance ($\sigma_{R_0}^2$) of R_0, the number of infections caused by an infected individual over the course of their infection.

First, if we assume a fixed infection period of D days, then we see that the total number of infections from an individual with infectiousness β is the sum of D draws form a Poisson distribution with mean β. Therefore,

$$p(R_0;\beta) = \frac{e^{-D\beta}(D\beta)^{R_0}}{R_0!}.$$

Thus across the entire population, with infectiousness described by an arbitrary distribution $p(\beta)$, the distribution of R_0 is simply $p(R_0) = \int_0^\infty d\beta p(\beta)p(R_0;\beta)$. From this distribution we can calculate the mean and variance of R_0.

$$\begin{aligned}
\mu_{R_0} &= \sum_{R_0=0}^{\infty} p(R_0)R_0 \\
&= \int_0^\infty d\beta p(\beta)e^{-D\beta}\sum_{R_0=0}^{\infty}\frac{(D\beta)^{R_0}}{R_0!}R_0 \\
&= \int_0^\infty d\beta p(\beta)D\beta \\
\mu_{R_0} &= D\mu_\beta
\end{aligned}$$

$$\begin{aligned}
\sigma_{R_0}^2 &= \sum_{R_0=0}^{\infty} p(R_0)(R_0-\mu_{R_0})^2 \\
&= \int_0^\infty d\beta p(\beta)e^{-D\beta}\sum_{R_0=0}^{\infty}\frac{(D\beta)^{R_0}}{R_0!}(R_0-D\mu_\beta)^2 \\
&= \int_0^\infty d\beta p(\beta)\left[D^2(\beta-\mu_\beta)^2 + D\beta\right] \\
\sigma_{R_0}^2 &= D^2\sigma_\beta^2 + D\mu_\beta
\end{aligned}$$

Combining these results we see that the duration of infection dispersion parameter in terms of the daily infection rate is

$$\frac{\mu_{R_0}^2}{\sigma_{R_0}^2} = \frac{\mu_\beta^2}{\sigma_\beta^2 + \frac{\mu_\beta}{D}}.$$

From this expression, it becomes apparent that while the two dispersion parameters are not exactly equivalent, in our case they are very nearly equal since $\sigma_\beta^2 \gg \mu_\beta/D$. If we let $D = 14$ days and calculate the duration of infection dispersion parameter we obtain $\mu_{R_0}^2/\sigma_{R_0}^2 = 0.093$, as compared to our value of 0.096.

One can be more careful and let the infectious period be described by an arbitrary distribution $p(D)$ with mean μ_D and variance σ_D^2. With this setup, the duration of infection dispersion gains an extra term in the denominator:

$$\frac{\mu_{R_0}^2}{\sigma_{R_0}^2} = \frac{\mu_\beta^2}{\sigma_\beta^2 + \left(\frac{\sigma_D}{\mu_D}\right)^2 (\mu_\beta^2 + \sigma_\beta^2) + \frac{\mu_\beta}{\mu_D}}.$$

If we use $\mu_D = 13.4$ days (https://www.ncbi.nlm.nih.gov/pmc/articles/PMC7409948/) and conservatively estimate $\sigma_D \sim 5$ days, then the dispersion parameters remain almost unchanged.

We would also like to make the point that our primary result is not a numerical value for the dispersion parameter, but the Lorenz curve relationship (Eq. (9) and Fig. 3 of the manuscript). This relation is based on the daily dispersion parameter, and allows one to obtain results for the percentage of cases caused by (say) the 20% of most infectious cases. Our results for the Lorenz curve are consistent with other studies, while providing more quantitative detail. Thus, the question about how the dispersion parameter is defined does not affect our primary finding.

Reviewer 2 Comment:

The authors point out that their estimates provide a lower bound on σ_k use this to identify prevalent superspreading throughout the USA. Given that superspreading of SARS-CoV-2 is generally accepted and the effort taken by the authors to quantify the effects of different assumptions, it might be more useful to use this methods of measuring superspreading to identify a range of possible values of σ_k based on plausible assumptions, that is, identify both lower and upper bounds on σ_k, and compare this to the existing estimates in the literature.

Response:

Reviewer 2 provides the insightful suggestion that our analysis would be strengthened if we provide an upper bound for σ_β. We have made some effort to do this, which we discuss below. We would also like to point out that, while the prevalence of superspreading of SARS-CoV-2 is indeed "generally accepted" at this point (as the reviewer points out), this was much less the case in July of last year, when we first wrote this paper and submitted it for review!

In an effort to address the referee's recommendation that we provide an upper-bound estimate for σ_β, we make use of the analysis in Appendix S4 regarding the percentage of detected cases. There we show that the variance of new cases per current infection should decrease with the number of detected cases as:

$$\mathrm{Var}\left(\frac{\Delta I_{\mathrm{det}}}{I_{\mathrm{det}}}\right) = \frac{\mu_\beta + \mu_\beta^2(1 - \overline{p_{\mathrm{det}}}) + \overline{p_{\mathrm{det}}}\sigma_\beta^2}{I_{\mathrm{det}}}. \tag{1}$$

We have added a discussion of how we could use this expression to estimate a possible range of values for the variance in infectiousness to Appendix S4. If we use the percentage of asymptomatic cases, currently estimated at 40% (https://www.cdc.gov/coronavirus/2019-ncov/hcp/planning-scenarios-h.pdf), as a rough (conservative) estimate for the mean percentage of undetected cases, then μ_β remains unchanged while σ_β increases from 0.59 cases/day to 0.75 cases/day. This change in the variance corresponds to a significant increase in superspreading, as the percentage of new infections cause by the top 5% of infectious cases rises from 61.7% to 74.0%. While this exercise provides some insight into how large σ_β could be, it is not a rigorous upper bound. Firstly, there remains significant uncertainty in the percentage of asymptomatic cases as estimates range from 8.2% to 75% (https://journals.plos.org/plosone/article?id=10.1371/journal.pone.0241536). Additionally, there remain other complications, such as incubation period variance and cross-county interactions, which would increase the variance further. Since these details are difficult to quantify analytically,

our method is best reserved for producing a lower bound on the variance based on an early-time time series of the case count, rather than producing a full rigorous confidence interval.

We could, of course, compute a confidence interval for σ_β using the statistical uncertainty of our inference. However, given the large systematic issues that affect the upper bound (including detection rates, cross-county interactions, variation in incubation period, etc.), we prefer to present our result only as a lower bound on the true value.

Reviewer 2 Comment:

The authors address many potential complications that could impact their findings, including varying testing rates in different counties (Appendix S4). A further complication would be differentially increasing testing rates, where some counties ramped up testing faster than others. Similarly, the estimates might be impacted by day of week patterns in reported cases, where weekly cases often show cyclical patterns indicating that testing and reporting of confirmed cases is variable based on the day of the week. While I doubt either of these would substantially affect the major findings of the paper, some discussion of the potential impact of this would be useful.

Response:

Reviewer 2 raises an important question regarding the impact of both differential time dependence in testing rates across the counties and cyclical patterns in data reporting. To address this concern, we need to slightly rethink the way in which $\overline{p_{\text{det}}}$ appears in Eq. 1. Since we are calculating the variance in $\Delta I_{\text{det}}/I_{\text{det}}$ between various counties at a fixed I_{det}, rather than a fixed time, $\overline{p_{\text{det}}}$ will be calculated as an average in detection rates across various counties when they reach I_{det} cases. This will also lead to an average over various days of the week. Therefore, the cyclic weekly reporting trends will be averaged out across various counties since when a county reaches I_{det} is independent of the day of the week.

For the issue of differentially increasing detection rate, we can consider what happens when each county, i, has some dependence of the detection rate on time. For convenience, we can write the detection rate as $p_{i,\text{det}}(I_{\text{det}})$. Then, regardless of the variance in the detection rates across the counties, the variance in $\Delta I_{\text{det}}/I_{\text{det}}$ only depends on the mean detection rate. Furthermore, since we perform our analysis with the assumption that $p_{\text{det}} = 1$, any decrease in this quantity would lead to an increase in the calculated σ_β. Intuitively, this can be understood by realizing that the smaller average detection rate implies a larger effective population of infected individuals. We know that a larger effective population leads to a decreased $\text{Var}(\Delta I_{\text{det}}/I_{\text{det}})$. Therefore, to observe this large variance in new infections per case, the underlying variance in infectiousness must increase to overcome this suppression (represented by the $\overline{p_{\text{det}}}$ in from of the σ_β^2 term in Eq. (1)).

In short, we expect that cyclical patterns in detection rate should have minimal impact on our findings, while spatial variations in the time-dependent detection rates only increase the variance σ_β^2, reaffirming that our estimate is a lower bound. We have added additional clarification in Appendix S4 to more clearly discuss this crucial point raised by the reviewer.

Reviewer 2 Comment:

Figure 1: Further details should be provided as to the set of counties and time periods included in part B of the figure.

Response:

The caption on Fig. 1 of the original text has been amended to clearly state that part B contains the data from all of the counties included in the analysis.

Reviewer 2 Comment:

Line 94: The authors state that the recovery period for COVID-19 is 14 days, but other literature suggest that viral shedding may decline and seroconversion may occur 7 days after symptom onset (e.g. Wölfel et al. 2020 Virological assessment of hospitalized patients with COVID-2019. Nature. https://doi.org/10.1038/s41586-020-2196-x). While this is negligible in the scheme of affecting the portion of the population susceptible, it nevertheless would be more accurate to provide a range here.

Response:

A range and citation have been added to Line 94 to accurately convey the most up to date range for duration of infectiousness. This will help clarify that a fixed infection period is not required for our study to be robust.

Reviewer 2 Comment:

Figure 2: The caption for could be made clearer by indicating that the blue points are bucketed data where buckets are determined by confirmed cases. Additionally, clarification should be made as to how the number of confirmed cases was determined (at the end of the 14 day period?). Finally, the y-axis in Figure 2 needs additional labels to be informative.

Response:

The caption for Fig. 2 in the original text has been updated to more clearly state the method for calculating the blue data points.

Reviewer 2 Comment:

I would strongly encourage the authors to make the code publicly available.

Response:

We have added a note on line 183 stating that the code is available here.

Finally, due to an indexing discrepancy, the counties quoted in the caption of Fig. 1 were incorrect. The caption has been updated to reflect the correct counties.

Thank you again for your consideration,
Calvin Pozderac and Brian Skinner
